# Barriers and motivational factors towards physical activity in daily life living with COPD – an interview based pilot study

**DOI:** 10.1080/20018525.2018.1484654

**Published:** 2018-07-06

**Authors:** Elisabeth Bomholt Østergaard, Sajitha Sophia Sritharan, Anne Dal Kristiansen, Pernille Maja Thomsen, Anders Løkke

**Affiliations:** a Department of Physiotherapy Aarhus, VIA University College, Aarhus, Denmark; b Department of Respiratory Diseases and Allergy, Aarhus University Hospital, Aarhus, Denmark; c Department of Training, Hjørring Municipality, Hjørring, Denmark; d Physiotherapy Department, Elderly Centre Møllestien, Aarhus, Denmark

**Keywords:** Chronic obstructive pulmonary disease (COPD), rehabilitation, physical activity in daily life (PADL), exercise, general practitioners, barriers, enablers, motivation, information, physical and psychological benefits

## Abstract

**Background:** In Denmark, few people with Chronic Obstructive Pulmonary Disease (COPD) engage in physical activity although it is evident that pulmonary rehabilitation has positive effects on physical activity, dyspnoea, anxiety, fatigue and quality of life.

**Objective:** The purpose of this pilot study was to explore why people with COPD do not engage in physical activity and to explore motivational factors and barriers towards physical activity. Furthermore, to explore the role general practitioners have in this matter.

**Design:** We conducted fieldwork among five people with COPD and three general practitioners using qualitative semi-structured interviews. We made a thematic analysis and our analytical perspective was based on The Health Belief Model and Self Determination Theory.

**Results:** Findings revealed that people with COPD was not active because they did not receive the necessary information from the general practitioners about the benefits of physical activity neither about the negative consequences of an inactive lifestyle. Motivational factors were knowledge about COPD and benefits of physical activity. Experiencing the benefits on their own bodies, feeling that it was not dangerous to feel breathless and being successful coping with breathlessness were motivational. Functional tests like walking tests were very important and motivational for the participants because they outlined the progress achieved during activity and provided evidence of progress that was easy to comprehend compared with spirometry tests. General practitioners did not inform about the benefits of physical activity because they felt that medication was more important than physical activity and that people with COPD would not be motivated to be active.

**Conclusions:** The main reason for people with COPD not being physically active in our study was lack of sufficient information from their general practitioners. This study described some barriers, enablers and motivational factors for a physically active lifestyle and the general practitioners’ role in this. Thus, it is important that people with COPD receive early information about physical activity - and it should start with the general practitioners, who are the gate keepers in the health care system. We recommend that lung function test results are never used as a single indicator of disease progression and that more focus should be paid to functional tests like The Shuttle Walking Test or The Six Minute Walking Test.Further studies to identify barriers to, and facilitators for referral people with COPD to physical activity in daily life from the perspective of Danish general practitioners are required.

## Introduction

Worldwide chronic obstructive pulmonary disease (COPD) constitutes a growing public health challenge [1] and is the fourth leading cause of death in the world although it is both preventable and treatable [2]. COPD is also common in Denmark, and the incidence is very high compared with the rest of the World. The disease is one of the most frequent causes of all medical hospitalisations and costs related to COPD hospitalisations in Denmark are enormous and there are major human and socioeconomic consequences associated with COPD [3] as well as a high frequency of physical inactivity and immobilisation [4]. In Denmark the death rate is very high and Danish women with COPD have the highest death rate in Europe [3].

The evidence for the benefits of physical activity and pulmonary rehabilitation for people with COPD is very strong. As long ago as 1991, Brian L. Tiep[5] stated:

Pulmonary rehabilitation is the only approach to chronic lung disease short of lung transplantation that improves the long-term outlook for these patients. Thus, despite having an irreversible disease, people with COPD and restrictive lung diseases can benefit substantially from pulmonary rehabilitation. They learn to accept and tolerate dyspnoea, overcome anxiety and depression while building endurance, strength, and confidence thus becoming more independent and mobile. (5:591)

1996–2015 meta-analyses from Lacasse et al. [6–9] and McCarthy et al. [10] have added to the body of evidence reporting that pulmonary rehabilitation involving exercise therapy/physical activity as one of the components relieves dyspnoea and fatigue, improves emotional functions, enhances the sense of individual control, benefits health-related quality of life and increases exercise and functional capacity. However, in spite of strong evidence, it appears to be very difficult to recruit people with COPD to attend pulmonary rehabilitation [11], and participation in pulmonary rehabilitation is reported to be uniformly low all over the world [12]. A systematic review underlines that physical activity is not widely implemented in clinical practice [13]. Two questions appears: ‘Why do people with COPD not participate in pulmonary rehabilitation and why is physical activity not widely implemented in clinical practice?’

In 2013 the definition of pulmonary rehabilitation was revised [14,15] and the *new* ATS/ERS definition of pulmonary rehabilitation is:

Pulmonary rehabilitation is a comprehensive intervention based on a thorough patient assessment followed by patient-tailored therapies which include, but are not limited to, exercise training, education and behavior change, designed to improve the physical and psychological condition of people with chronic respiratory disease and to promote the long-term adherence to health-enhancing behaviors. (12:1170,13:e16)

So The ATS/ERS statement on pulmonary rehabilitation emphasizes the importance of promoting an adaptive behaviour change, especially collaborative self-management attempting to gain a higher level of physical activity in *daily life* (PADL) [14,15]. Collaborative self-management strategies promote self-efficacy – confidence in successfully managing one’s health – which are central in acquiring new adaptive behaviour [15]. Nevertheless The ATS/ERS statement on pulmonary rehabilitation also states that exercise training remains the ‘cornerstone of rehabilitation’ (12:1170).

Many patients with COPD have no previous experience of exercise training and they describe exercise training, as something unknown and unimportant for them [16]. However it does not need to be exercise training and it does not need to take place in a gym or in a fitness centre, the important thing is to do activities on daily basis – optimally generating breathlessness, e.g. Borg Scale 16–17 (15:393). Singh et al [14] highlights remembering walking because it is easy, cheap and meaningful – additional to supporting participants to be actively involved in their disease management, and a need to explore patients misconceptions about their disease and the value of rehabilitation.

Today PADL is in focus, and especially how to increase PADL is one of the most difficult puzzles in rehabilitation of people with COPD [17]. We have to ask ourselves: How do we get higher levels of PADL? Does pulmonary rehabilitation result in higher level of PADL? It is obvious that a more individually tailored and comprehensive intervention is required to change human behaviour to achieve a higher level of PADL [18].

The aim of this pilot study was to explore why people with COPD are not physically active in their daily life and to explore motivational factors and barriers to promote physical activity. Furthermore, to get an insight into the role of the general practitioners because they are the gate keepers in the Danish health care system. The target group of this study is health professionals and in particular the general practitioners because they most often have the initial contact with people with COPD.

## Materials and methods

### Design

We conducted qualitative field studies in Denmark from 2013 to 2015 consisting of semi-structured interviews with five people with COPD (Appendix 1) and with three general practitioners (Appendix 2). The primary investigator (author EBØ^1^) and the secondary investigators (author ADK^3^, PMT^4^), all physiotherapists, carried out these interviews. In addition, author EBØ^1^ is also Master in the Anthropology of Health. We also participated, mostly passively [19], in the conversation (Danish) in a Facebook group for people with COPD. The other authors (SSS^2^, AL^5^) from Department of Respiratory Diseases and Allergy worked as sparring partners.

Interviews with one of the people with COPD and the three general practitioners, and participation in the Facebook group conversation took place in 2013 (author EBØ^1^), and more interviews with four people with COPD took place in 2015 (authors ADK^3^, PMT^4^).

The reason for using a qualitative research design was to explore the experiences of people with COPD and their meeting with the Danish healthcare system. The reason for interviewing the general practitioners was to get an insight in their way of managing the meeting with people with COPD. By participating mostly passively in the Facebook group conversation, we ‘listened’ to the daily conversation without disturbing. We were like a fly on the wall, an unnoticed observer and listener, trying to catch some important points. However, two times we had to participate more actively in the conversation by telling about the benefits of physical activity, in the particular situations we felt that it would have been inappropriate not to do so.

### Participants

Five people diagnosed with COPD were recruited. We wanted both participants who were physically active and some who were physically inactive. Posters put up in six senior community centres resulted in one participant, contact to a big physiotherapeutic clinic resulted in one informant, contact to a university hospital (physiotherapy department) resulted in one participant and posters put up in two Facebook groups for people with COPD resulted in two participants. No participants were recruited from the specific Facebook group in which we participated/carried out our field study. We selected people diagnosed 4–10 years ago and therefore had COPD-specific experience; their level of activity ranged from no physical activity to regular activity on daily basis. The three general practitioners were recruited from Central Denmark Region. From a telephone list we selected from different places – one from a big city, one from the neighbourhood of a big city and one from a smaller city in the countryside. They were given the option of conducting the interview face-to-face or by telephone. The general practitioners represented themselves.

### Data collection

The interviews with people with COPD were audio recorded with a MP3 or an iPhone and transcribed field notes were made. Four of these participants were interviewed in their homes to ensure a safe environment and to relate the interview specifically to the daily living of each participant. One of these participants did not want to be interviewed at home so this interview took place at VIA University College in Aarhus, Denmark. Two interviews with general practitioners were conducted by telephone and one interview was conducted at the general practitioners’ own home. Field notes were made.

### Data analysis

We transcribed the interviews transforming spoken into written language [20] and we made a thematic analysis [21]. Our analytical perspective was theories about The Health Belief Model (HBM) [22] and Self-Determination Theory (SDT) [23]. The HBM is a psychological theory from the 1950s attempting to explain and predict health behaviour. There are six constructs in the model which affect health behaviour: *perceived susceptibility, perceived severity, perceived benefits, perceived barriers, cues to action and self-efficacy*. The model proposes that peoples’ belief about health issues, perceived benefits of action and barriers to action, and self-efficacy explain the commitment to health-promoting behaviour. A stimulus or cue to action must be present to trigger that health-promoting behaviour [22].

The SDT is a theory of motivation describing several types and degrees of motivation. SDT is, among others, divided into two theories: Cognitive Evaluation Theory (CET) and Organismic Integration Theory (OIT). CET comprises intrinsic motivation based on own satisfactions of behaving. OIT concerns extrinsic motivation and explains the different ways a person’s behaviour can be motivated extrinsically [23].

### Ethics

Our study complied with The Declaration of Helsinki [24]. We anonymised the data and names of the participants to protect individual confidentiality [24,25]. We informed the participants about the aim of the study, the method, anonymity and our professional confidentiality and the participants gave written informed consent [25]. We refer to participants as ‘people with COPD’ using people-first language [26–30]. The aim is to emphasize a holistic perspective, to signal superior importance of the person rather than the disease by describing what the person has not what the person is [25]. Our work was approved by The Central Denmark Region Committee on Health Research Ethics, Stoltenberg 26, Postboks 21, 8800 Viborg, Denmark.

## Results

### Description of participants

The participants consisted of four women and one male with moderate (*n* = 2) and severe (*n* = 3) COPD, aged between 39 and 75 years. Four out of the five participants were diagnosed with COPD more than 5 years ago and had been physically active. Two of the participants had comorbidities that could influence their physical activity. Table 1 shows participant characteristics.

**Table 1.** Characteristics of the participants – people with COPD.

	Participant I Lis (pseudonym)	Participant II Helle (pseudonym)	Participant III Inger (pseudonym)	Participant IV Per (pseudonym)	Participant V Helga (pseudonym)
Age (years)	53	39	62	68	75
Sex	Female	Female	Female	Male	Female
Self-reported severity of COPD forced expiratory volume (FEV1)	Moderate (54%)	Moderate (68%)	Severe (30–40%)	Severe (42%)	Severe (37%)
Years since diagnosed with COPD	4	7	8	10	6
Physical activity	No physical activity	Moderate physical activity, e.g. using e-bike	Moderate physical activity, e.g. water aerobics once a week	Regular physical activity on every day basis, team exercise training, walking etc.	Regular physical activity on every day basis, team exercise training, walking etc.
Comorbidity	Prolapsed intervertebral disc, operated 4 times		Arthrosis ankles, knees and hips, diabetes		
Interviewed by	Author ADK^3^ 2015	Author PMT^4^ 2015	Author PMT^4^ 2015	Author ADK^3^ 2015	Author EBØ^1^ 2013

### A major transition in life

The participants experienced the diagnosis of COPD as a great shock and a major change, followed by a period of psychological crisis, ending with acceptance of life with COPD. In a ritual perspective, the participants moved from separation through liminality to integration [31,32].

Participant IV told that he was very sad in the beginning and thought that life had come to an end:

In my view, it was a death sentence, but it turned out that there is a life even though you have COPD!

The participants felt that they in their daily life had to cope with limitations and sometimes fear related to living with COPD. They perceived they had less freedom and ability to be spontaneous.

Participant I told that before the COPD diagnosis, she would go to the city by herself but now she could not because she was afraid of getting an attack of breathlessness, even though she had never experienced it.

I fear getting an attack of breathlessness when I am alone.

### Quality of life

In general, the most important aspect was having an independent daily life and to maintain and hopefully increase the level of activity. Participant II mentioned daily activities such as shopping, cooking and going for a walk as aspects increasing quality of life. Participant IV emphasised that physical activity especially together with others improved his quality of life:

You leave the team training with renewed energy and your mood improves by 20, 30, 40, 50 degrees

Participant V prioritised physical training with a physiotherapist twice a week:

I get more energy when I exercise.

I just have to be able to go for a walk!

For Participant III it was important to take her time and to avoid breathlessness by not planning too many things. Quality of life was having a good time, reading a book, painting and getting a cup of coffee. She did not prioritize physical activity:

Smoking is my pleasure, understand it or not.

### Lack of early information about the benefits of physical activity

General practitioners act as gatekeepers in the Danish healthcare system as they decide whether patients can be treated in general practice or should be referred for further diagnostics. The participants were asked what kind of information they received from their general practitioner and the health care system after getting the diagnosis of COPD.

The participants experienced that they did not receive any early information about the benefits of physical activity. They were not informed by their general practitioners about physical activity or about the opportunities for improving physical capacity neither for coping with COPD nor about the negative consequences of an inactive lifestyle.

Participant V explains:

Why is it that the general practitioner does not know more about getting people (with COPD) started with training? They should know, because it is the first step we take, contacting the general practitioner, it is our own doctor. I think they do not know. I didn’t get any information, not until I got to the lung clinic.

After a long time, often several years, when getting in contact with the lung clinic and the rehabilitation programme, the participants experienced being informed about the benefits of physical activity.

One of the participants reported the general practitioner being supportive and talking about coping with COPD.

### Barriers

The participants perceived different barriers to physical activity and behavioural changes.

Not knowing the benefits of physical activity and being afraid of breathlessness during exercise were barriers.

Anxiety could also be a barrier. Participants II and III were sometimes anxious about being too breathless, which limited their activities. Participant II expressed it in this way:

Well, there is an implicit threshold and if you cross it then something can happen, so you don’t go, I dare not.

Participant IV told that he could not control his breathlessness when he was pushing himself to the limit, but he was not scared about it anymore.

Participant V told:

I just breathe until I cannot continue, and it is the same procedure when I am cleaning the house, because you have to vacuum and wash the floor, and then I sit down and when I am breathing more calmly again I continue.

### Lack of rehabilitation possibilities for people with COPD

The participants did not feel comfortable about exercising in fitness centres; they needed their own facilities.

Participant I pointed out that she had no fitness centre within walking distance as it was unmanageable going by bus or other public transportation.

The participants more frequently experienced exacerbations leading to rapid decondition and prolonged recovery. Other disabilities like arthrosis as well as muscular pain and/or weakness also contributed substantially to exercise limitations.

### Motivational factors

The participants pointed to several essential, motivational factors. Information about COPD, benefits of physical activity and that breathlessness during exercise is not dangerous, but beneficial.

Participant V did not receive information until 3 years after the diagnosis when she came to the lung clinic:

They explained everything about COPD

I did everything. I stopped smoking and I trained.

Participant V clearly remembered that the doctor at the lung clinic said, *‘It isn’t dangerous to get breathless!’* and it meant a lot to her. The physical activity led her to the next motivational factor because she began to feel the benefits on her own body:

You get more energy, when you do the physical exercises! …. I could very much feel that it worked.

In general, the participants highlighted the benefits of physical exercise as a great motivational factor. Participant II expressed it very clearly:

The better fitness, the less breathlessness, the more I endure the stronger muscles, the easier I can do the tasks and daily activities.

Functional tests like walking tests to measure physical fitness and strength motivated the participants as well as promoting optimism and hope for the future. They found functional tests more useful and easier to comprehend than lung function tests.

Participant IV:

Going from two to 6–8 shuttles in the walking test, well it clearly tells me the progress.

Team exercise motivated due to recognition and understanding of the common challenges living with COPD. They felt safe and accepted and were not met with condemnation.

Professional supervision was considered optimal. For some a fitness instructor was fine but others felt safer with a physiotherapist or another health care professional. Being physically active with family or friends resulted in increasing the effort.

Others were motivated by competitions (e.g. soccer or beating your own record when walking), fear of death, dependency of oxygen etc.

### General practitioners

None of the three general practitioners informed the participants about physical activity. They diagnosed and prescribed medical treatment. When asked if they informed about physical activity, one general practitioner stated:

No, the most important is medicine!

Another general practitioner told that she did not inform the patients because she expected it would not have any consequences. She stated that people with COPD generally sat in a chair at home and smoked. She expected that it would be difficult to motivate them, like all of us in general:

They should be physically active – we all should.

### Participation in a closed group for people with COPD

When new members entered the closed COPD group they typically asked what to do. They had been diagnosed and now they felt frightened, scared, sad, depressed, disillusioned, uncertain about the future and afraid that life was coming to an end very soon. They did not know what to do and how to manage living with COPD and they had got no information about the benefits of physical training.

## Discussion

In Denmark, and most likely in most of the industrialised world, only few people with COPD engage in physical activity even though there is evidence that pulmonary rehabilitation has positive effects on dyspnoea, anxiety, fatigue and quality of life. This study explores reasons for not engaging in physical activity in daily life among people with COPD in Denmark as well as motivational factors to promote physical activity through a qualitative approach with interviews with five people with COPD and with three general practitioners.

The people with COPD who participated in our study experienced that they did not get information from their general practitioners about COPD, the benefits of physical activity or about the negative consequences of being inactive. It took several years before they felt adequately informed about the benefits of physical activity and no longer feared breathlessness during exercise.

As being diagnosed with COPD was associated with a period of sadness and uncertainty, maybe even depression, some information may have been provided by general practitioners but not understood until later in the process of adapting to life with COPD.

Not knowing about the benefits of physical activity was a considerable barrier. Breathlessness sometimes resulted in anxiety and it was a process to learn how to cope with this. Lack of COPD-specific activity courses was also a barrier because it was unpleasant to be an outsider exercising together with healthy people. Comorbidities and exacerbations were challenging because of accompanying physical deterioration and difficulties recovering. This led to lack of motivation because the achieved results from physical activity were lost and restart exercising seemed to be without purpose. One participant also felt that transportation was a major barrier.

In the perspective of HBM [22] experiences of benefits and threats are important preconditions to behavioural changes. Self-efficacy is important for lifestyle and behavioural changes [33].

Motivational factors for being physically active were first of all information about COPD and the benefits of physical activity. It was also important to know that breathlessness during exercise is not dangerous, but actually the opposite.

Feeling the benefits of physical activity and success coping with breathlessness were strong intrinsic motivational factors. In a SDT perspective [23], participant V at first was extrinsically motivated which secondly led to intrinsic motivation when she felt the benefits of physical activity on her own body.

COPD-specific activity supervised by health care professionals, preferable a physiotherapist, had very high priority because it allowed the participants to feel safe and train harder.

Functional tests like walking tests were very important for the participants because they outlined the progress achieved during activity and provided evidence of progress that was easy to comprehend compared with spirometry tests. This was a very useful motivational factor. We therefore recommend that lung function test results are never used as a single indicator of disease progression and that more focus should be paid to functional tests like The Shuttle Walking Test [34] or The Six Minute Walking Test [35].

Quality of life in general was improved by physical activity, good mood and capability to do desired things like walking or visiting others, shopping and cleaning the house. For one of the participants physical activity was not a priority and smoking provided quality of life.

None of the three general practitioners in this study informed people with COPD about physical activity or referred them to a programme. They believed that people with COPD would not be motivated to be physically active, so they did not feel it was important to inform them about activity as a part of COPD treatment. They believed that medical treatment was more important.

Supporting this, a larger study from Australia [12] found that 10 of 12 general practitioners interviewed had not directly referred a person with COPD for pulmonary rehabilitation, and barriers among the general practitioners were low knowledge of rehabilitation management of people with COPD.

Thus, information about the positive effects of physical activity is basic in making a positive change for the individual regarding maintaining and improving physical capacity and quality of life, and for society with a potentially positive impact on the socioeconomic burden of COPD. Johnston et al. (12:323) conclude that referral by the general practitioners to pulmonary rehabilitation programmes is a crucial step in achieving implementation of this high-evidence guideline recommendation in the care of people with COPD, and specific strategies to integrate pulmonary rehabilitation into daily workflow of general practitioners are required.

Benefits for people with COPD and society could possibly be achieved if general practitioners in addition to providing medical treatment also focused more on informing people with COPD of the benefits of physical activity. When benefits are so evident and among other things increases functional capacity and individual control, they can lead to a more active daily life with less admission to hospital for exercabation of symptoms [12] and probably less pathological changes in the lungs – and possibly prevent disease progression.

The target group of this study is health professionals and in particular the general practitioners because they most often have the initial contact with people with COPD.

## Limitations

The main limitation of this pilot study is the small sample size consisting of people with moderate and severe COPD, which limits the generalizability.

The three general practitioners interviewed might not be representative of the general point of view among general practitioners.

Every aspect of COPD has not been thoroughly examined in this study as socioeconomic factors, the extent of social network as well as possible discrepancies between living in the city and in the countryside, use of daily oxygen or not, differences in severity of COPD and differences in the burden of comorbidities were not considered. This could be explored further in future studies.

## Conclusions

The main reason for people with COPD not being physically active in our study was lack of sufficient information from their general practitioners. This study described some barriers, enablers and motivational factors for a physically active lifestyle and the general practitioners’ role in this. It would be relevant to further explore how people newly diagnosed with COPD experience the level and quality of information about the benefits of physical activity and the negative consequences of an inactive lifestyle.

This pilot study was conducted as the precursor of a larger study to gather knowledge and information in this area within a larger sample of people with COPD and general practitioners to further explore and validate barriers and enablers to physical activity. Especially, further studies to identify barriers to, and facilitators for referral people with COPD to physical activity in daily life from the perspective of Danish general practitioners are required.

## Clinical messages

- People with COPD do not engage in physical activity, mainly because they do not get sufficient information from their general practitioners.
- We strongly recommend general practitioners to inform people with COPD about the benefits of physical activity, because it is basic for changing lifestyle into a more active daily life, and we strongly recommend not only to prescribe medicine.
- We recommend that the lung function test is never used as a single test when COPD because it cannot show improvements in physical capacity and therefore can generate demotivation for changing daily life.
- We recommend that more focus should be paid to functional tests like, e.g. The Shuttle Walking Test [34] or The Six Minute Walking Test [35] in COPD, because they can assess and show improvements in physical capacity and therefore generate motivation for further activity in daily life.

## Appendix 1

**Table:** Interview guide(translated from Danish)**Overview of semi-structured interview guide to patients with COPD**

***Research questions***	***Interview questions***
What is quality of life for the patients?	**Daily life and quality of life**: • What is important for you in your daily life?
	• What gives you quality of life?
	• How does the disease affect you and you daily life/quality of life (limitations)?
What kind of information do general practitioners give the patients about the disease, and the patients’ own opportunities to change the severity of the disease?	**Information from the general practitioner**: • Which information did you get from your general practitioner about the disease?
	• Which examinations did you go through? What did the general practitioner tell about the results? What did the results tell you?
	• What did the general practitioner say that you could do about the disease?
	• What is your knowledge about physical activity? (Knowledge from the general practitioner or others)
How does the patients’experience from physical activity affect their current level of physical activity?	**Experience with physical activity**: • How would you characterise your level of physical activity now and in the past?
	• What kind and to what extent are you physically active?
	• How is your experience with physical activity?
Which factors influence the patients’ current behaviour (activity/no activity) based on the Health Belief Model?	**Reasons to be physically active or not** • Perceived susceptibility and severity • How much does your disease affect you psychologically? What are your thoughts about the disease?
	• How do you think your disease will affect you in the future?
	• How does it affect you that the disease cannot be cured? Perceived benefits and barriers
	• Which thoughts do you have about doing physical activity with COPD?
	• What keeps you from doing physical activity? What are the reasons for you to be physically active? What could stop you from being physically active?
	• What are pros and cons of your current level of activity?
	• What are the pros and cons of a high level of physical activity?
	• Self-efficacy and cues to action How is your experience on being able to control the disease? Experiences from physical activity.
	• What do you think it takes to improve your condition? Which factors can be influenced by yourself?
	• How do you see your own opportunities for getting more physically active?
	• Do you know other patients with COPD? Are they physically active? What are their experiences?
What is needed to increase the patients’ motivation to be more physically active based on the Self Determination Theory?	**Motivation** • To what extent do you feel motivated? Ask based on the scale.
	• What is needed for you to start doing physical activity?
	• What could help you being more physically active? E.g. reward, praise, satisfaction, fun, avoid guilt, goals, team activity?
	• What do you achieve from physical activity? What is your motivation for physical activity? Why do you choose that type of physical activity? What is the reason to continue being active?

## Appendix 2

Semi-structured interview guide for general practitioners (translated from Danish)

1. What do you do when a patient with lung problems comes to you?
2. What do you do if you suspect a patient has COPD or the patient got diagnosed with COPD?
3. Do you inform the patient about the importance of physical activity?
4. Do you inform the patient about physical activity and the effect they will get from it?

The answers from general practitioners

**GENERAL PRACTITIONER 1**

When a patient with lung problems approaches me, I do two things:

1. Diagnose the patient
2. Prescribe medicine

I do not inform the patients about physical activity or refer them to a specialist in pulmonary medicine. I think most of the general practitioners do not do this. I think that when the patients get admitted to the hospital due to an exacerbation, then they will probably be referred to a specialist.

**GENERAL PRACTITIONER 2**

If a person comes with lung problems, I will stethoscope the person, take an electrocardiogram, perform a pulmonary function test and take blood samples. I will try to diagnose and treat the patient.

I will treat them with medicine and inhalations. Maybe I will refer the patient to the department of pulmonary diseases.

I am not good at telling the patients to be physically active. They are just sitting at home and smoking and it is hard to motivate them. They should be physically active – we all should.

**GENERAL PRACTITIONER 3**

If I suspect a patient has COPD I perform a pulmonary function test and give them medicine.

I do not inform the patients about the importance of physical activity, because the most important thing is the medication.
